# Association of Variation in Consultant Use Among Hospitalist Physicians With Outcomes Among Medicare Beneficiaries

**DOI:** 10.1001/jamanetworkopen.2019.21750

**Published:** 2020-02-21

**Authors:** Jennifer P. Stevens, Laura A. Hatfield, David J. Nyweide, Bruce Landon

**Affiliations:** 1 Center for Healthcare Delivery Science, Beth Israel Deaconess Medical Center, Boston, Massachusetts; 2 Division for Pulmonary, Critical Care, and Sleep Medicine, Department of Medicine, Beth Israel Deaconess Medical Center, Boston, Massachusetts; 3 Department of Health Care Policy, Harvard Medical School, Boston, Massachusetts; 4 Center for Medicare & Medicaid Innovation, Centers for Medicare & Medicaid Services, Baltimore, Maryland; 5 Division of General Medicine, Department of Medicine, Beth Israel Deaconess Medical Center, Boston, Massachusetts

## Abstract

**Importance:**

Evidence is lacking on the consequences of high rates of inpatient consultation.

**Objective:**

To examine outcomes and resource use of patients cared for by hospitalists who use more inpatient consultation than their colleagues.

**Design, Setting, and Participants:**

A retrospective cohort study of medical admissions to hospitalists among fee-for-service Medicare beneficiaries was conducted. Hospitalist consultation tendency was identified from January 1, 2013, to December 31, 2014; admissions were calculated in 2013; and outcomes were measured in 2014. Data were analyzed from January 31, 2017, to May 9, 2019. A total of 711 654 admissions with patients receiving care from 14 584 hospitalists at 737 hospitals were included.

**Exposure:**

Admission to high-consulting hospitalists, considered to be those who were in the top 25% of the distribution of consulting frequency at their own hospital (adjusted for patient case mix).

**Main Outcomes and Measures:**

Outcomes included length of stay, Medicare Part B inpatient charges, discharge destination, all-cause 7- and 30-day readmissions, 90-day outpatient specialist visits, and 30-day mortality.

**Results:**

The 711 654 hospital admissions included 408 489 women (57.4%); mean (SD) age of the population was 80 (8.5) years. Length of stay of patients cared for by high-consulting hospitalists was longer compared with other hospitalists (adjusted incidence rate ratio, 1.04; 95% CI, 1.03-1.05). The admissions resulted in a mean of $137.91 (95% CI, $118.89-$156.93) more in Medicare Part B charges and were less likely to end with the patient going home (adjusted odds ratio [aOR], 0.96; 95% CI, 0.94-0.98) compared with patients cared for by other hospitalists in the cohort. Patients cared for by high-consulting hospitalists also were 7% more likely than patients cared for by other hospitalists to see an outpatient specialist at 90 days (aOR 1.07; 95% CI, 1.05-1.09), with no significant differences in 30-day mortality (aOR 1.01, 95% CI, 0.98-1.03) or readmissions (7-day readmissions: aOR 1.01; 95% CI, 0.98-1.03; 30-day readmissions: aOR, 1.01; 95% CI, 0.99-1.03).

**Conclusions and Relevance:**

Hospitalists who obtain consultations more than their colleagues at the same institution were associated with greater use of health care resources without apparent mortality benefit. Further investigation should identify whether reducing high rates of consultation can reduce resource use without harming patients.

## Key Points

**Question:**

Are hospitalized Medicare beneficiaries who are exposed to higher rates of specialty consultation associated with outcomes such as greater resource use, length of stay, readmissions, and mortality?

**Findings:**

In this cohort study of 711 654 inpatient medical admissions, hospitalists who used specialty consultation more than their colleagues at the same institution used more resources without a difference in patient mortality. Compared with patients treated by other hospitalists, the patients of high-consulting hospitalists had longer lengths of stay, were less likely to go home, and were more likely to see a specialist within 90 days after discharge, but there was no significant difference in their mortality at 30 days or their likelihood of all-cause readmission.

**Meaning:**

A decrease in the frequency of specialty consultation may be an opportunity for hospitals to reduce complexity and costs in patient care without adversely affecting patients.

## Teaser

This cohort study examines whether higher rates of specialty consultation are associated with outcomes of hospitalized US Medicare beneficiaries.

## Introduction

As US health care spending continues to increase more quickly than inflation, attention has focused on reducing delivery of health care services that do not benefit patients.^1,2,3,4^ However, because most hospitals in the United States are paid via a fixed diagnosis-adjusted reimbursement per admission, little research has assessed low-value care during hospitalizations. Hospital care represents the largest share of health care spending, despite many hospitals operating with negative or low margins.^5,6^ Hospitals may need to provide more efficient care to remain financially viable.^7^ Many hospital services are used without significant benefit to patients, including excessive inpatient telemetry for patients with low risk of acute coronary syndrome,^8,9^ daily chest radiographs in the intensive care unit,^10,11^ and routine laboratory testing for clinically stable patients.^12^ Yet, curtailing use of such services may yield only incremental savings.^13^

One opportunity to curb excessive use of services that may add to costs without providing significant benefit to patients, considered low-value care for patients, is the use of specialty care for hospitalized patients. Inpatient specialty consultation, that is, engaging a specialist during the care of a hospitalized patient, is prevalent: Medicare beneficiaries average 1 to 3 consultations per admission.^14,15^ The substantial variation in physicians’ use of consultation, however, is not fully explained by hospital characteristics or patient need.^15^ While specialty consultation brings additional clinical expertise and access to procedures,^16,17,18,19^ the use of consultations may increase resource use and have negative clinical consequences. For example, specialist involvement can increase length of stay to accommodate evaluation and testing by the new physician team, and testing or procedures that ultimately are not useful also carry risks for overdiagnosis or complications. Specialists may reflexively recommend ongoing outpatient care after discharge, potentially increasing costs, testing, and travel for patients. Specialty consultation also may lead to low-value radiologic or laboratory studies or risky additional procedures both in the hospital and after discharge. Thus, reducing consultation use beyond clinical need might be an important target for hospitals seeking to become more efficient and reduce low-value care for patients.

Studying the benefits and risks of consultation is fraught with confounding. Patients with many specialty consults are generally sicker, and the marginal consequences of consultation may be outweighed by these patient differences. Moreover, practice styles and specialty care use vary widely within and between hospitals.^15^ We sought to identify variation in specialty consultation that was independent of patients’ medical needs, health outcomes, and hospital differences. We examined the nearly random assignment of hospitalized patients to an attending hospitalist as a source of plausibly exogenous variation in patients’ exposure to inpatient consultation. We characterized hospitalists relative to their colleagues in the same hospital and compared the outcomes of admissions to high consulting hospitalists with the outcomes of the remaining hospitalists. We hypothesized that admissions of patients receiving care from high-consulting hospitalists would be associated with greater resource use without appreciable differences in patient outcomes.

## Methods

### Study Population and Data Source

We used administrative data from Medicare to analyze admissions of fee-for-service Medicare beneficiaries during 2013 and 2014. Using the Medicare Beneficiary Summary File, we included beneficiaries aged 66 years and older during the year of admission who were continuously enrolled in Parts A and B without end-stage renal disease. We excluded those enrolled in Medicare Advantage at any point during the year. We limited our sample to hospitalizations with hospitalist attendings for the 25 most prevalent medical diagnosis related groups (top 25 medical DRGs) (eTable 1 in the Supplement) to capture the most common medical admissions. We restricted institutions to nonfederal (ie, excluding critical access, Indian Health Service, and Veteran Affairs hospitals) acute care hospitals located in the 50 states and the District of Columbia that were large enough to measure variability in consultation behavior across hospitalists (≥250 beds and ≥8 hospitalists with ≥10 admissions). Medical admissions from January 1, 2013, to December 31, 2014, were included. Hospitalist consultation tendency was identified using admissions in 2013, and outcomes were measured in 2014. Data analysis was performed from January 31, 2017, to May 9, 2019. This study followed the Strengthening the Reporting of Observational Studies in Epidemiology (STROBE) reporting guideline for cohort studies. Our study was reviewed and deemed exempt with waiver of informed consent as a study conducted using existing publicly available data by the institutional review board at the Beth Israel Deaconess Medical Center, Boston, Massachusetts.

### Identifying Hospitalists and Classifying Hospitalist Consultation Tendency

We defined hospitalists as generalist physicians with at least 100 claims in 2013, of which at least 90% were for inpatient care.^20^ Each top 25 medical DRG admission was assigned a single attending hospitalist. Each hospitalist was assigned to the hospital at which the plurality of their 2013 and 2014 admissions were billed. We restricted participation to hospitalists with at least 10 admissions at their assigned hospital in 2013.

We estimated hospitalist consultation tendency using admissions in 2013. Admissions were assigned to hospitalists based on the attending physician assigned in the Part A claim.^21^ We computed the number of consultations per admission as the number of unique medical specialties billed per admission, minus 1 to account for the hospitalist. We fit a negative binomial regression model for the number of consultations per admission that included fixed effects for DRGs to account for patient case mix. The residuals from this model represent the relative number of consultations for each admission compared with the mean for admissions with the same DRG.

We determined the mean of these residuals for each hospital and each hospitalist. Then, for each hospitalist, we subtracted their hospital’s mean residual from their own mean residual to yield a relative measure of the hospitalist’s tendency to use consultation compared with other hospitalists at the same institution. We defined high-consulting hospitalists as those in the top 25% of the distribution. As a sensitivity check, we also compared hospitalist characterizations based on 2013 admissions with how those same hospitalists would have been characterized based on 2014 admissions.

Because we were interested in the patient outcomes associated with exposure to high consultation use, we sought to capitalize on the pseudorandom assignment of patients to hospitalists by admission date and the relative stability of hospitalists’ consultation tendencies over time. We present the between-hospital variation in consultation tendency as well, as most of the variation occurs between hospitals rather than within hospitals; however, the large degree of between-hospital variation may also be associated with variation in hospital resources, specialty availability, and region of the country.

### Study Variables

For length of stay and cost, all patient outcomes were measured during admissions in 2014 to further eliminate confounding by indication that may be associated with high consultation use. We defined length of stay as the date of discharge minus the date of admission plus 1 such that each admission had a length of stay of at least 1 day. For cost, we summed Part B–allowed costs between the admission and discharge dates of the hospitalization to quantify the additional costs to the Medicare program beyond the fixed cost per DRG of the hospitalization.

Using the discharge destination variable in the inpatient file, we dichotomized discharge into the patient’s home vs all other destinations and restricted to patients alive through discharge. We defined outpatient specialist use as any outpatient physician claim from a medical specialist within 90 days of discharge. We measured this outcome only on patients who were alive through 90 days after discharge. We restricted this measure to a patient’s first hospitalization of the year and to those who were alive through discharge to calculate all-cause readmissions at 7 and 30 days after discharge. We identified all-cause mortality at 30 days after admission using the Master Beneficiary Summary File, again restricting to a patient’s first hospitalization.

### Patient and Hospital Control Variables

Beneficiary characteristics included age (in 5-year increments), race and ethnicity (defined from the Chronic Conditions Warehouse race variable as black, Hispanic, non-Hispanic white, Asian, and other), female sex, dual eligibility for Medicare and Medicaid, Medicare coverage through disability, chronic conditions defined by Chronic Conditions Warehouse chronic condition ever flags, severity of the DRG (defined based on whether the DRG code included complications and comorbidities using the admission DRG severity index), whether the admission occurred on a weekend, and, for some outcomes, inpatient mortality. Hospital characteristics from the 2013 American Hospital Association survey included teaching status (major teaching [Council of Teaching Hospitals members], minor teaching [any hospital with residents or medical students], and nonteaching), rural location, US Census region (Northeast, South, Midwest, and West), percentage of patients admitted to the intensive care unit, and mean hospital hierarchal condition categories score.^22^ The last 2 measures capture hospital-level resource use and case mix complexity. Scores are determined by the percentage of intensive care unit admissions from 2013 MedPAR files and hospital-level means of merged hierarchal condition categories scores for each admitted beneficiary.

### Statistical Analysis

We compared the unadjusted outcomes of admissions to high-consulting hospitalists vs the remaining admissions using χ^2^ tests for categorical outcomes and 2-tailed, paired *t* tests or Wilcoxon rank sum tests for continuous outcomes. We fit generalized linear models with distributions and link functions appropriate to each outcome. Specifically, for binary outcomes (readmission, discharge home, mortality, and outpatient specialty care), we used binomial distributions with logit links; for the count outcome of length of stay, we used a negative binomial distribution with a log link; and for the continuous outcome of cost, we used a gaussian distribution with an identity link. In these models, we clustered residuals at the hospital level and adjusted for all available beneficiary and hospital characteristics. *P* < .05 was considered statistically significant. All analyses were performed using SAS Enterprise Guide, version 7.15 (SAS Institute Inc).

## Results

We analyzed 711 654 admissions between January 1, 2014, and December 31, 2014, with patients cared for by 14 584 hospitalists at 737 hospitals. Patient and hospital characteristics are described in Table 1; in summary, mean (SD) patient age was 80 (8.5) years and 408 489 were women (57.4%). Hospitalists obtained a mean (SD) of 1.2 (1.2) consultations per admission. High-consulting hospitalists used 1.4 (1.3) consults per admission compared with 1.1 (1.2) consults per admission for remaining hospitalists.

**Table 1.** Patient and Hospital Characteristics Between High-Consulting Hospitalists and Others With Absolute Differences, 2014

Characteristic	%			Absolute Difference	*P* Value for Difference
	All Patients	High-Consulting Hospitalists	Other Hospitalists		
Total No.	711 654	171 235	540 419	NA	NA
Consultations/admission, mean (SD), No.	1.2 (1.2)	1.4 (1.3)	1.1 (1.2)	0.3	<.001
**Patient**					
Demographic characteristics					
Age, mean (SD), y	80.1 (8.5)	80.1 (8.5)	80.1 (8.5)	0	.01
Women	57.4	57.3	57.5	0.2	.34
Non-Hispanic white	83.8	83.1	84.1	1.0	<.001
Black	11.2	11.7	11.1	0.6	<.001
Hispanic	1.6	1.8	1.5	0.3	<.001
Asian	1.4	1.6	1.3	0.3	<.001
Other race	2.0	1.8	2.0	0.2	<.001
Medicaid and Medicare dual eligibility	25.8	26.4	25.6	0.8	<.001
Disabled	15.7	15.6	15.8	0.2	.10
Admission					
Admitted on weekend	25.5	25.5	25.5	0	.60
DRG severity					
Lowest	28.6	28.2	28.7	0.5	<.001
Medium	41.0	40.8	41.1	0.3	.05
Highest	30.4	31.0	30.3	0.7	<.001
Inpatient death	3.8	3.9	3.8	0.1	.36
Comorbidities					
Cardiac	87.6	88.4	87.3	1.1	<.001
Renal	69.9	70.5	69.7	0.8	<.001
Hematologic-oncologic	24.6	24.8	24.5	0.3	.01
Neurologic	58.2	58.9	58.0	0.9	<.001
Endocrine	93.7	94.1	93.6	0.5	<.001
Rheumatic	72.1	72.6	72.0	0.6	<.001
Pulmonary	61.0	61.7	60.7	1.0	<.001
**Hospital**					
Case mix of hospital^a^					
Hospital mean HCC score					
Quartile 1 (<1.61)	8.0	6.4	8.5	2.1	<.001
Quartile 2 (1.61-1.76)	29.5	25.1	30.9	5.8	<.001
Quartile 3 (1.76-1.91)	37.2	37.8	37.0	0.8	<.001
Quartile 4 (>1.91)	25.3	30.6	23.6	7.0	<.001
Hospital mean ICU admissions/y					
Quartile 1 (0)	5.0	5.4	4.8	0.6	<.001
Quartile 2 (0-0.10)	37.9	38.8	37.6	1.2	<.001
Quartile 3 (0.10-0.16)	38.8	37.4	39.3	1.9	<.001
Quartile 4 (>0.16)	18.4	18.4	18.3	0.1	<.001
Ownership					
Government, nonfederal	8.3	7.0	8.7	1.7	<.001
Not-for-profit	83.0	84.9	82.3	2.6	<.001
For-profit	8.8	8.1	9.0	0.9	<.001
Teaching status^b^					
Major teaching hospital	25.8	30.0	24.5	5.5	<.001
Minor teaching hospital	19.0	18.6	19.1	0.5	<.001
Region					
Rural	0.1	0.3	0.1	0.2	<.001
Northeast	24.5	27.0	23.7	3.3	<.001
Midwest	21.3	21.1	21.3	0.2	.13
South	39.0	38.8	39.0	0.2	.13
West	15.3	13.1	15.9	2.8	<.001

Abbreviations: DRG, diagnosis related group; HCC, hierarchal condition categories; ICU, intensive care unit; NA, not applicable.

^a^ Scores are determined by the percentage of ICU admissions from 2013 MedPAR files and hospital-level means of merged hierarchal condition categories scores for each admitted beneficiary. For the intensive care unit use, values were quartile 1, 0.0; quartile 2, 0.096; and quartile 3, 0.16. For the hierarchal condition category scores, values were quartile 1, 1.61; quartile 2, 1.76; and quartile 3, 1.91.

^b^ Major comprised Council of Teaching Hospitals members; minor, any hospital with residents or medical students.

Consultation tendency was relatively stable over time between 2013 and 2014; approximately 7% of previously low-consulting hospitalists in 2013 became top-quartile consulting hospitalists in 2014, and 7% of high-consulting hospitalists in 2013 became low-consulting hospitalists in 2014. Hospitals varied in their mean use of inpatient consultations on the hospitalist services (Figure).

**Figure.**
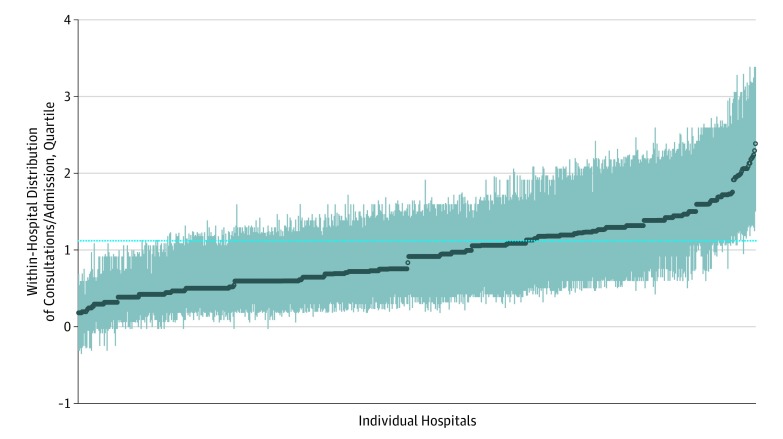
Within-Hospital Distribution of Residual Consultations per Admission Each line spans from a hospital's 25th to 75th percentile in the distribution of residual consults per admission (across hospitalists). The residuals come from the model fit to 2013 admissions, adjusted for diagnosis related group. The solid points mark the hospital medians. The national mean of 1.1 consultations per admission (horizontal dotted line) was added to each value to aid interpretation of the scale.

Characteristics of 2014 admissions to high-consulting hospitalists vs others are presented in Table 1. Patient demographic and admission characteristics for admissions to high-consulting hospitalists vs others were similar; however, high-consulting hospitalists cared for more nonwhite patients (16.9% vs 15.9%), more Medicaid dual-eligible patients (26.4% vs 25.6%), and more patients in the highest severity DRG (31.0% vs 30.3%). There was evidence of differential allocation of admissions to high-consulting hospitalists at some hospitals; although high-consulting hospitalists compose 25% of hospitalists in each hospital (by definition), they may care for more than 25% of admissions.

Patients cared for by high-consulting hospitalists had 4% longer lengths of stay (unadjusted mean: 6.1 vs 5.8 days; *P* < .001; adjusted incidence rate ratio, 1.04; 95% CI, 1.03-1.05) and more than 11% higher part B costs relative to patients cared for by their colleagues at the same institution (unadjusted mean: $1427 vs $1265; *P* < .001; adjusted difference: $137.91; 95% CI, $118.89-$156.93). Patients were 4% less likely to go home (unadjusted risk of going home: 41.5% vs 42.9%; *P* < .001; adjusted odds ratio [aOR], 0.96; 95% CI, 0.94-0.98) and 7% more likely to see a specialist at 90 days (unadjusted risk of specialist visit at 90 days: 66.5% vs 64.9%; *P* < .001; aOR, 1.07; 95% CI, 1.05-1.09) if they were cared for by high-consulting hospitalists, with no significant difference in readmission rates at 7 days (aOR, 1.01; 95% CI, 0.98-1.03) or 30 days (aOR, 1.01; 95% CI, 0.99-1.03). In addition, there was no significant difference in 30-day mortality for patients cared for by high-consulting hospitalists (unadjusted mean: 10.3% vs 10.3%; *P* = .62; aOR, 1.01; 95% CI, 0.98-1.03). Unadjusted analyses are presented in Table 2; adjusted analyses are presented in Table 3. The full models are available in eTables 2-8 in the Supplement.

**Table 2.** Unadjusted Outcomes of Admission to High-Consulting Hospitalists vs Other Hospitalists, 2014

Outcome	High-Consulting Hospitalists	Other Hospitalists	Absolute Difference	*P* Value for Difference
Length of stay, mean (SD), d	6.1 (4.5)	5.8 (4.1)	0.3	<.001
Spending, mean (SD), $	1427 (1250)	1265 (1106)	162	<.001
Discharge home, %	41.5	42.9	1.4	<.001
Readmission, %				
7 d	6.0	5.9	0.1	.05
30 d	17.5	17.0	0.5	<.001
Visit to specialist at 90 d, %	66.5	64.9	1.6	<.001
30-d mortality, %	10.3	10.3	0	.62

**Table 3.** Results From Regression Models Comparing Adjusted Outcomes of Admissions to High-Consulting Hospitalists vs Other Hospitalists, 2014

Adjusted Outcome	Top Quartile of Hospitalists vs Other Hospitalists, Adjusted Odds Ratio (95% CI)^a^
Length of stay^b^	1.04 (1.03-1.05)
Spending, mean, $^c^	137.9 (118.9-156.9)
Discharge home	0.96 (0.94-0.98)
Readmission, d	
7	1.01 (0.98-1.03)
30	1.01 (0.99-1.03)
Visit to specialist at 90, d	1.07 (1.05-1.09)
30-d mortality	1.01 (0.98-1.03)

Abbreviation: OR, adjusted odds ratio.

^a^ Top quartile defined as hospitalists who were in the top 25% of the distribution of consulting frequency at their own hospital (adjusted for patient case mix).

^b^ Data are presented as incidence rate ratio (95% CI).

^c^ Data are presented as mean (95% CI).

## Discussion

Our results suggest an association between high-consulting hospitalists and the use of more resources without clear benefit to patients, which appears to be true even after controlling for consultation tendencies at the hospital level. Patients who were cared for by high-consulting hospitalists stayed longer in the hospital, were less likely to go home, and were more likely to see an outpatient specialist within 90 days of discharge but showed no evidence of differences in readmission rates compared with patients cared for by other hospitalists. There was no significant difference in survival at 30 days for patients between groups after controlling for mean consultation use at the hospital level.

Prior work examining the patient-level consequences of inpatient specialty use has focused solely on the expertise provided by specific specialties.^23,24,25,26,27^ For instance, Sellier et al^28^ noted that adherence to infectious disease consultants’ recommendations was associated with lower inpatient mortality and earlier clinical improvement. However, findings by Jena and colleagues^29^ raised the concern that patients may not always routinely benefit from specialty consultation; they found that patients admitted with cardiac diagnoses did comparably better during times of major cardiology conferences when only junior cardiologists were available. Although we did not find harm to patients in the form of increased mortality or readmissions with exposure to more consultations, we found increases in resource use, including both costs incurred during the hospitalization and increased subsequent use of outpatient specialty care.

Our approach did not allow us to distinguish the exact point at which consultations are no longer useful, and this threshold likely is not uniform across different diagnoses and specialties consulted or across different hospitals. Thus, although our results do not highlight a group of patients actively harmed by potentially high rates of inpatient consultation, we have identified a group of physicians who apparently provide relatively more resource-intensive care, which could be a target for education or other interventions by hospitals. Thus, a decrease in frequent consultation, particularly for diagnoses or questions in which there is likely to be little change in management, may be an opportunity for hospitals to reduce complexity and costs in patient care without adversely affecting patients. It is likely that there may be underuse of consultation by some hospitalists, but our research suggests that at least some portion of consultations represent overuse. Further study distinguishing by type of consultation (eg, procedural consultations, specific specialty consultations) may be helpful in clarifying the heterogeneity of associations with various types of inpatient consultation.

Our study design used the pseudorandom assignment of patients to hospitalists by admission date to identify the benefit or harm from frequent use of specialty consultation. However, we found that some observable hospital-level variables, such as teaching status and geographic location, were not completely balanced. We attribute this imbalance to differential shares of admissions going to high-consulting hospitalists in some hospitals (eg, Northeast hospitals and teaching hospitals). High-consulting hospitalists may use specialty care at their institutions potentially to fill clinical knowledge gaps or, alternatively, compensate for excessive patient caseload and offload clinical work. The lack of balance of some hospital-level variables in our treatment assignment may lend further credence to our hypothesis that the highest-consulting hospitalists at times may be using consultation to accommodate heavy workloads.

### Limitations

Our study has several limitations. First, we used administrative data, which lack the clinical nuance of medical records. More specifically, we did not explore other patient outcomes beyond mortality, such as patient satisfaction. Second, as with any observational study, our findings may be biased by unmeasured confounders, such as patterns in how certain admissions are assigned to hospitalists, and so cannot claim that hospitalist consultation levels cause certain outcomes. However, we note the relative stability of our findings between the unadjusted and adjusted models and that any unmeasured confounder in the adjusted models would have to be uncorrelated with the patient- and hospital-level variables included, adding further confidence to our results. Third, we sought to study the patient-level outcome of high-consulting hospitalists, which fails to indicate the benefit or harms associated with individual consultations. Fourth, we did not explore additional features of the hospitalists that might be associated with specialty use; these may represent unmeasured confounders with some of the outcomes (eg, length of stay), although less likely with others (eg, outpatient specialty use at 90 days). Fifth, we assigned admissions to hospitalists based on the Part A designation of the attending physician, which may fail to accurately attribute the consultation tendency or patient outcomes to the correct physician. However, the Part A attending is defined as the physician who has “overall responsibility for the beneficiary’s care”^21^ and is therefore likely to be most responsible for clinical decision-making. Furthermore, any misclassification would likely bias our findings toward the null. Sixth, we did not account from where a patient was admitted (which is not accurately recorded in administrative data), which may influence whether a patient could be discharged home. Seventh, we necessarily limited the sample to hospitals with a high volume of admissions and multiple hospitalists to measure the variability in consultation tendency for the most common medical reasons for hospitalization. Therefore, our findings may not be generalizable to smaller hospitals.

## Conclusions

This study found that care provided by high-consulting hospitalists may be associated with increased resource use without clear benefit to patients. Our findings suggest that consultation may be one area in which hospitals or health systems seeking to decrease costs, such as under risk-based models of care, could focus their attention. Alternative strategies to minimize overuse of consultation may have the potential to yield additional savings to hospitals and added benefits to patients, particularly among elderly patients for whom attending outpatient visits may be difficult or returning home may be a priority.

## Notes

Supplement. **eTable 1.** Most Common Medical DRGs **eTable 2.** Complete Multivariable Model for High-Consulting Hospitalist Models, Top Quartile Versus All Others, With Outcome of Length of Stay **eTable 3.** Complete Multivariable Model for High-Consulting Hospitalist Models, Top Quartile Versus All Others, With Outcome of Part B Spending **eTable 4.** Complete Multivariable Model for High-Consulting Hospitalist Models, Top Quartile Versus All Others, With Outcome of Discharge Home **eTable 5.** Complete Multivariable Model for High-Consulting Hospitalist Models, Top Quartile Versus All Others, With Outcome of Readmission at 7 Days **eTable 6.** Complete Multivariable Model for High-Consulting Hospitalist Models, Top Quartile Versus All Others, With Outcome of Readmission at 30 Days **eTable 7.** Complete Multivariable Model for High-Consulting Hospitalist Models, Top Quartile Versus All Others, With Outcome of Visit to a Specialist at 90 Days **eTable 8.** Complete Multivariable Model for High-Consulting Hospitalist Models, Top Quartile Versus All Others, With Outcome of Mortality at 30 Days
