# Supplementary material for: Association of Variation in Consultant Use Among Hospitalist Physicians With Outcomes Among Medicare Beneficiaries
Source: JAMA Netw Open. 2020 Feb 21;3(2):e1921750. doi: 10.1001/jamanetworkopen.2019.21750 (PMC7043199; doi:10.1001/jamanetworkopen.2019.21750)
Supplement: Supplement. — eTable 1. Most Common Medical DRGs eTable 2. Complete Multivariable Model for High-Consulting Hospitalist Models, Top Quartile Versus All Others, With Outcome of Length of Stay eTable 3. Complete Multivariable Model for High-Consulting Hospitalist Models, Top Quartile Versus All Others, With Outcome of Part B Spending eTable 4. Complete Multivariable Model for High-Consulting Hospitalist Models, Top Quartile Versus All Others, With Outcome of Discharge Home eTable 5. Complete Multivariable Model for High-Consulting Hospitalist Models, Top Quartile Versus All Others, With Outcome of Readmission at 7 Days eTable 6. Complete Multivariable Model for High-Consulting Hospitalist Models, Top Quartile Versus All Others, With Outcome of Readmission at 30 Days eTable 7. Complete Multivariable Model for High-Consulting Hospitalist Models, Top Quartile Versus All Others, With Outcome of Visit to a Specialist at 90 Days eTable 8. Complete Multivariable Model for High-Consulting Hospitalist Models, Top Quartile Versus All Others, With Outcome of Mortality at 30 Days [file jamanetwopen-3-e1921750-s001.pdf]

Supplementary Online Content

Stevens JP, Hatfield LA, Nyweide DJ, Landon B. Association of variation in consultant use among hospitalist physicians with outcomes among Medicare beneficiaries. *JAMA Netw Open*. 2020;3(2):e1921750. doi:10.1001/jamanetworkopen.2019.21750

eTable 1. Most Common Medical DRGs

eTable 2. Complete Multivariable Model for High-Consulting Hospitalist Models, Top Quartile Versus All Others, With Outcome of Length of Stay

eTable 3. Complete Multivariable Model for High-Consulting Hospitalist Models, Top Quartile Versus All Others, With Outcome of Part B Spending

eTable 4. Complete Multivariable Model for High-Consulting Hospitalist Models, Top Quartile Versus All Others, With Outcome of Discharge Home

eTable 5. Complete Multivariable Model for High-Consulting Hospitalist Models, Top Quartile Versus All Others, With Outcome of Readmission at 7 Days

eTable 6. Complete Multivariable Model for High-Consulting Hospitalist Models, Top Quartile Versus All Others, With Outcome of Readmission at 30 Days

eTable 7. Complete Multivariable Model for High-Consulting Hospitalist Models, Top Quartile Versus All Others, With Outcome of Visit to a Specialist at 90 Days

eTable 8. Complete Multivariable Model for High-Consulting Hospitalist Models, Top Quartile Versus All Others, With Outcome of Mortality at 30 Days

This supplementary material has been provided by the authors to give readers additional information about their work.

© 2020 Stevens JP et al. *JAMA Network Open.*

eTable 1. Most common medical DRGs

DRG	DRG full name
291	HEART FAILURE & SHOCK W MCC
292	HEART FAILURE & SHOCK W CC
293	HEART FAILURE & SHOCK W/O CC/MCC
194	SIMPLE PNEUMONIA & PLEURISY W CC
193	SIMPLE PNEUMONIA & PLEURISY W MCC
195	SIMPLE PNEUMONIA & PLEURISY W/O CC/MCC
192	CHRONIC OBSTRUCTIVE PULMONARY DISEASE W/O CC/MCC
190	CHRONIC OBSTRUCTIVE PULMONARY DISEASE W MCC
191	CHRONIC OBSTRUCTIVE PULMONARY DISEASE W CC
872	SEPTICEMIA OR SEVERE SEPSIS W/O MV 96+ HOURS W/O MCC
871	SEPTICEMIA OR SEVERE SEPSIS W/O MV 96+ HOURS W MCC
690	KIDNEY & URINARY TRACT INFECTIONS W/O MCC
689	KIDNEY & URINARY TRACT INFECTIONS W MCC
392	ESOPHAGITIS, GASTROENT & MISC DIGEST DISORDERS W/O MCC
391	ESOPHAGITIS, GASTROENT & MISC DIGEST DISORDERS W MCC
308	CARDIAC ARRHYTHMIA & CONDUCTION DISORDERS W MCC
309	CARDIAC ARRHYTHMIA & CONDUCTION DISORDERS W CC
310	CARDIAC ARRHYTHMIA & CONDUCTION DISORDERS W/O CC/MCC
377	G.I. HEMORRHAGE W MCC
378	G.I. HEMORRHAGE W CC
379	G.I. HEMORRHAGE W/O CC/MCC
066	INTRACRANIAL HEMORRHAGE OR CEREBRAL INFARCTION W/O CC/MCC
064	INTRACRANIAL HEMORRHAGE OR CEREBRAL INFARCTION W MCC
065	INTRACRANIAL HEMORRHAGE OR CEREBRAL INFARCTION W CC
640	NUTRITIONAL & MISC METABOLIC DISORDERS W MCC
641	NUTRITIONAL & MISC METABOLIC DISORDERS W/O MCC
683	RENAL FAILURE W CC
682	RENAL FAILURE W MCC
684	RENAL FAILURE W/O CC/MCC
177	RESPIRATORY INFECTIONS & INFLAMMATIONS W MCC
179	RESPIRATORY INFECTIONS & INFLAMMATIONS W/O CC/MCC
178	RESPIRATORY INFECTIONS & INFLAMMATIONS W CC

© 2020 Stevens JP et al. *JAMA Network Open.*

603	CELLULITIS W/O MCC
602	CELLULITIS W MCC
281	ACUTE MYOCARDIAL INFARCTION, DISCHARGED ALIVE W CC
282	ACUTE MYOCARDIAL INFARCTION, DISCHARGED ALIVE W/O CC/MCC
280	ACUTE MYOCARDIAL INFARCTION, DISCHARGED ALIVE W MCC
811	RED BLOOD CELL DISORDERS W MCC
812	RED BLOOD CELL DISORDERS W/O MCC
390	G.I. OBSTRUCTION W/O CC/MCC
388	G.I. OBSTRUCTION W MCC
389	G.I. OBSTRUCTION W CC
299	PERIPHERAL VASCULAR DISORDERS W MCC
300	PERIPHERAL VASCULAR DISORDERS W CC
301	PERIPHERAL VASCULAR DISORDERS W/O CC/MCC
552	MEDICAL BACK PROBLEMS W/O MCC
551	MEDICAL BACK PROBLEMS W MCC
393	OTHER DIGESTIVE SYSTEM DIAGNOSES W MCC
394	OTHER DIGESTIVE SYSTEM DIAGNOSES W CC
395	OTHER DIGESTIVE SYSTEM DIAGNOSES W/O CC/MCC
287	CIRCULATORY DISORDERS EXCEPT AMI, W CARD CATH W/O MCC
286	CIRCULATORY DISORDERS EXCEPT AMI, W CARD CATH W MCC
637	DIABETES W MCC
639	DIABETES W/O CC/MCC
638	DIABETES W CC
948	SIGNS & SYMPTOMS W/O MCC
947	SIGNS & SYMPTOMS W MCC
373	MAJOR GASTROINTESTINAL DISORDERS & PERITONEAL INFECTIONS W/O CC/MCC
371	MAJOR GASTROINTESTINAL DISORDERS & PERITONEAL INFECTIONS W MCC
372	MAJOR GASTROINTESTINAL DISORDERS & PERITONEAL INFECTIONS W CC
202	BRONCHITIS & ASTHMA W CC/MCC
203	BRONCHITIS & ASTHMA W/O CC/MCC
056	DEGENERATIVE NERVOUS SYSTEM DISORDERS W MCC
057	DEGENERATIVE NERVOUS SYSTEM DISORDERS W/O MCC

© 2020 Stevens JP et al. *JAMA Network Open.*

eTable 2. Complete multivariable model for high-consulting hospitalist models, top quartile versus all others, with outcome of length of stay.

				95% confidence interval		
			High-consulting	Lower bound	Upper bound	p-value for difference
IRR for LOS			1.0402	1.0328	1.0476	<.0001
Patient	Demographic characteristics	Age 71-75 (REF Age < 70)	0.9942	0.988	1.0004	0.0654
		Age 76-80	0.9935	0.9875	0.9995	0.0339
		Age 81-85	0.9868	0.9805	0.9931	<.0001
		Age 86-90	0.9695	0.9629	0.9761	<.0001
		Age 91-95	0.9497	0.9421	0.9572	<.0001
		Age > 95	0.919	0.9098	0.9283	<.0001
		Women (REF men)	0.9976	0.9939	1.0013	0.201
		Black (REF white race)	1.0061	0.995	1.0173	0.2837
		Hispanic	0.9651	0.9439	0.9867	0.0017
		Asian	0.9985	0.9783	1.0191	0.8823
		Other race	1.0112	0.9941	1.0285	0.1997
		Medicaid dual-eligible	1.0569	1.0505	1.0633	<.0001
		disabled	1.0167	1.0108	1.0225	<.0001
	Admission characteristics	Admitted on weekend	0.9826	0.9789	0.9863	<.0001
		Medium DRG severity (REF low DRG severity)	1.2957	1.2887	1.3028	<.0001
		Highest DRG severity	1.5154	1.5052	1.5256	<.0001
		Inpatient mortality	1.109	1.0952	1.1229	<.0001
		Cardiac comorbidities	1.0501	1.0439	1.0562	<.0001
		Renal comorbidities	1.1253	1.1206	1.13	<.0001
		Heme-onc comorbidities	1.0104	1.0065	1.0144	<.0001

© 2020 Stevens JP et al. *JAMA Network Open.*

		Neurologic comorbidities	1.0596	1.0547	1.0644	<.0001
		Endocrine comorbidities	0.9618	0.9551	0.9684	<.0001
		Rheumatologic comorbidities	1.0011	0.9969	1.0052	0.6147
		Pulmonary comorbidities	1.0281	1.0239	1.0322	<.0001
Hospital	Case mix of hospital	Q2 hospital mean HCC score (REF Q1, lowest, mean HCC score)	0.9915	0.9688	1.0147	0.4695
		Q3 hospital mean HCC score	1.0047	0.9825	1.0274	0.6785
		Q4 hospital mean HCC score	1.011	0.9876	1.0348	0.3598
		Q2 hospital mean ICU admissions per year (REF Q1, lowest, mean ICU admissions per year)	1.0164	0.9855	1.0483	0.3027
		Q3 hospital mean ICU admissions per year	0.9978	0.9669	1.0297	0.8889
		Q4 hospital mean ICU admissions per year	1.0264	0.9911	1.063	0.1449
	Ownership	Government, non-federal (REF not-for-profit)	1.0211	0.9929	1.0502	0.1443
		For-profit	1.0105	0.984	1.0377	0.4418
	Teaching status	Major teaching hospital (REF nonteaching hospital)	1.0514	1.0331	1.07	<.0001
		Minor teaching hospital	1.0134	0.9939	1.0333	0.1784

© 2020 Stevens JP et al. *JAMA Network Open.*

	Region	Rural (Ref urban)	0.9531	0.9347	0.9719	<.0001
		Northeast (REF West)	1.119	1.0928	1.1459	<.0001
		Midwest	1.0075	0.9874	1.028	0.4673
		South	1.0706	1.0502	1.0915	<.0001

© 2020 Stevens JP et al. *JAMA Network Open.*

eTable 3. Complete multivariable model for high-consulting hospitalist models, top quartile versus all others, with outcome of Part B spending.

				95% confidence interval		
			High-consulting	Lower bound	Upper bound	p-value for difference
GEE for Part B spending			137.91	118.89	156.93	<.0001
Patient	Demographic characteristics	Age 71-75 (REF Age < 70)	7.4081	-3.222	18.038	0.172
		Age 76-80	-0.746	-11.69	10.198	0.8937
		Age 81-85	-40.54	-52.51	-28.57	<.0001
		Age 86-90	-99.61	-113.6	-85.66	<.0001
		Age 91-95	-176.1	-192.7	-159.4	<.0001
		Age > 95	-247.4	-266	-228.8	<.0001
		Women (REF men)	-39.37	-45.34	-33.4	<.0001
		Black (REF white race)	-86.18	-107.3	-65.04	<.0001
		Hispanic	-39.64	-91.95	12.675	0.1375
		Asian	19.336	-15.84	54.514	0.2813
		Other race	72.845	13.589	132.1	0.016
		Medicaid dual-eligible	62.977	49.536	76.417	<.0001
		disabled	-3.034	-13.24	7.1731	0.5602
	Admission characteristics	Admitted on weekend	-22.85	-29.33	-16.37	<.0001
		Medium DRG severity (REF low DRG severity)	404.98	393.48	416.48	<.0001
		Highest DRG severity	638.01	619.74	656.28	<.0001
		Inpatient mortality	601.03	562.37	639.7	<.0001
		Cardiac comorbidities	171.5	163.03	179.97	<.0001
		Renal comorbidities	160.79	152.23	169.35	<.0001
		Heme-onc comorbidities	36.19	28.767	43.614	<.0001

© 2020 Stevens JP et al. *JAMA Network Open.*

		Neurologic comorbidities	123.01	114.09	131.93	<.0001
		Endocrine comorbidities	54.826	43.665	65.987	<.0001
		Rheumatologic comorbidities	73.983	66.409	81.557	<.0001
		Pulmonary comorbidities	0.025	-7.612	7.6622	0.9949
Hospital	Case mix of hospital	Q2 hospital mean HCC score (REF Q1, lowest, mean HCC score)	14.361	-43.22	71.945	0.625
		Q3 hospital mean HCC score	64.922	6.5233	123.32	0.0293
		Q4 hospital mean HCC score	187.85	122.71	252.99	<.0001
		Q2 hospital mean ICU admissions per year (REF Q1, lowest, mean ICU admissions per year)	-53.14	-148.4	42.139	0.2743
		Q3 hospital mean ICU admissions per year	-78.47	-172.9	15.993	0.1035
		Q4 hospital mean ICU admissions per year	-51.5	-154.5	51.483	0.327
	Ownership	Government, non-federal (REF not-for-profit)	-38.53	-105.4	28.304	0.2585
		For-profit	87.423	20.124	154.72	0.0109
	Teaching status	Major teaching hospital (REF nonteaching hospital)	12.314	-37.98	62.607	0.6313
		Minor teaching hospital	-47.11	-92.28	-1.933	0.041

© 2020 Stevens JP et al. *JAMA Network Open.*

	Region	Rural (Ref urban)	-325.9	-386.3	-265.5	<.0001
		Northeast (REF West)	73.137	6.0703	140.2	0.0326
		Midwest	-24.49	-80.64	31.652	0.3926
		South	-0.29	-56.19	55.607	0.9919

© 2020 Stevens JP et al. *JAMA Network Open.*

eTable 4. Complete multivariable model for high-consulting hospitalist models, top quartile versus all others, with outcome of discharge home

				95% confidence interval		
			High-consulting	Lower bound	Upper bound	p-value for difference
Odds ratio of discharge home			0.9614	0.9416	0.9816	0.0002
Patient	Demographic characteristics	Age 71-75 (REF Age < 70)	0.8681	0.8517	0.8847	<.0001
		Age 76-80	0.6914	0.6773	0.7058	<.0001
		Age 81-85	0.5188	0.5065	0.5313	<.0001
		Age 86-90	0.3792	0.3693	0.3894	<.0001
		Age 91-95	0.2926	0.2827	0.3029	<.0001
		Age > 95	0.2509	0.2392	0.2632	<.0001
		Women (REF men)	0.8907	0.8792	0.9024	<.0001
		Black (REF white race)	1.1523	1.1045	1.2022	<.0001
		Hispanic	1.7886	1.649	1.9399	<.0001
		Asian	1.6233	1.499	1.7579	<.0001
		Other race	1.326	1.2427	1.4147	<.0001
		Medicaid dual-eligible	0.5182	0.5049	0.5319	<.0001
		disabled	0.875	0.8575	0.8929	<.0001
	Admission characteristics	Admitted on weekend	0.986	0.9745	0.9975	0.0176
		Medium DRG severity (REF low DRG severity)	0.5894	0.5798	0.5992	<.0001
		Highest DRG severity	0.4077	0.3994	0.4162	<.0001
		Cardiac comorbidities	0.8749	0.8572	0.8929	<.0001
		Renal comorbidities	0.7196	0.7086	0.7308	<.0001
		Heme-onc comorbidities	0.9567	0.9432	0.9704	<.0001
		Neurologic comorbidities	0.4063	0.3996	0.4131	<.0001

© 2020 Stevens JP et al. *JAMA Network Open.*

		Endocrine comorbidities	1.1275	1.1015	1.154	<.0001
		Rheumatologic comorbidities	0.8979	0.8839	0.9121	<.0001
		Pulmonary comorbidities	0.9823	0.9677	0.9972	0.02
Hospital	Case mix of hospital	Q2 hospital mean HCC score (REF Q1, lowest, mean HCC score)	0.9896	0.8905	1.0998	0.8467
		Q3 hospital mean HCC score	0.9363	0.8415	1.0418	0.2267
		Q4 hospital mean HCC score	1.0391	0.9261	1.166	0.5136
		Q2 hospital mean ICU admissions per year (REF Q1, lowest, mean ICU admissions per year)	0.8956	0.7882	1.0177	0.0909
		Q3 hospital mean ICU admissions per year	0.8864	0.7793	1.0083	0.0667
		Q4 hospital mean ICU admissions per year	0.9405	0.8193	1.0796	0.3833
	Ownership	Government, non-federal (REF not-for-profit)	0.9798	0.8925	1.0756	0.6678
		For-profit	1.0777	0.9846	1.1796	0.1045
	Teaching status	Major teaching hospital (REF nonteaching hospital)	4.0055	0.9284	1.0889	0.8934
		Minor teaching hospital	1.0328	0.9573	1.1142	0.4048
	Region	Rural (Ref urban)	2.123	1.941	2.322	<.0001
		Northeast (REF West)	0.6741	0.6054	0.7506	<.0001

© 2020 Stevens JP et al. *JAMA Network Open.*

		Midwest	0.8399	0.7682	0.9182	0.0001
		South	0.9215	0.8467	1.0028	0.058

© 2020 Stevens JP et al. *JAMA Network Open.*

eTable 5. Complete multivariable model for high-consulting hospitalist models, top quartile versus all others, with outcome of readmission at 7 days

				95% confidence interval		
			High-consulting	Lower bound	Upper bound	p-value for difference
Odds ratio of readmission at 7 days			1.0088	0.9914	1.0266	0.3228
Patient	Demographic characteristics	Age 71-75 (REF Age < 70)	0.9053	0.8836	0.9275	<.0001
		Age 76-80	0.8371	0.8168	0.8579	<.0001
		Age 81-85	0.8015	0.78	0.8235	<.0001
		Age 86-90	0.7244	0.7047	0.7445	<.0001
		Age 91-95	0.6827	0.66	0.7063	<.0001
		Age > 95	0.6032	0.574	0.6339	<.0001
		Women (REF men)	0.9716	0.9572	0.9861	0.0001
		Black (REF white race)	1.0037	0.9798	1.0282	0.7659
		Hispanic	0.9931	0.9361	1.0535	0.817
		Asian	0.9852	0.923	1.0516	0.654
		Other race	0.9753	0.9256	1.0277	0.3491
		Medicaid dual-eligible	1.0728	1.0536	1.0923	<.0001
		disabled	1.0011	0.98	1.0228	0.9166
	Admission characteristics	Admitted on weekend	1.0011	0.986	1.0164	0.8885
		Medium DRG severity (REF low DRG severity)	1.1649	1.1438	1.1864	<.0001
		Highest DRG severity	1.3524	1.3268	1.3785	<.0001
		Cardiac comorbidities	1.589	1.5482	1.631	<.0001
		Renal comorbidities	1.8137	1.7814	1.8465	<.0001
		Heme-onc comorbidities	1.1772	1.1591	1.1956	<.0001
		Neurologic comorbidities	1.2221	1.2019	1.2427	<.0001

© 2020 Stevens JP et al. *JAMA Network Open.*

		Endocrine comorbidities	1.0385	1.0064	1.0717	0.0184
		Rheumatologic comorbidities	1.0744	1.0561	1.0931	<.0001
		Pulmonary comorbidities	1.2882	1.2681	1.3087	<.0001
Hospital	Case mix of hospital	Q2 hospital mean HCC score (REF Q1, lowest, mean HCC score)	1.0189	0.9779	1.0617	0.3717
		Q3 hospital mean HCC score	1.0665	1.0234	1.1114	0.0022
		Q4 hospital mean HCC score	1.1173	1.0713	1.1653	<.0001
		Q2 hospital mean ICU admissions per year (REF Q1, lowest, mean ICU admissions per year)	1.0397	0.9901	1.0918	0.1188
		Q3 hospital mean ICU admissions per year	1.0439	0.9937	1.0967	0.0878
		Q4 hospital mean ICU admissions per year	1.0582	1.0049	1.1143	0.0318
	Ownership	Government, non-federal (REF not-for-profit)	1.0366	0.9976	1.0771	0.0664
		For-profit	1.0826	1.0363	1.1309	0.0004
	Teaching status	Major teaching hospital (REF nonteaching hospital)	1.0682	1.0401	1.097	<.0001
		Minor teaching hospital	0.9729	0.9454	1.0013	0.0615
	Region	Rural (Ref urban)	1.3122	1.271	1.3548	<.0001
		Northeast (REF West)	1.0569	1.0221	1.093	0.0012

© 2020 Stevens JP et al. *JAMA Network Open.*

		Midwest	1.0379	1.0037	1.0733	0.0295
		South	1.0161	0.9867	1.0464	0.2862

© 2020 Stevens JP et al. *JAMA Network Open.*

eTable 6. Complete multivariable model for high-consulting hospitalist models, top quartile versus all others, with outcome of readmission at 30 days

				95% confidence interval		
			High-consulting	Lower bound	Upper bound	p-value for difference
Odds ratio of readmission at 30 days			1.0161	0.9956	1.037	0.125
Patient	Demographic characteristics	Age 71-75 (REF Age < 70)	0.9053	0.8836	0.9275	<.0001
		Age 76-80	0.8372	0.8169	0.858	<.0001
		Age 81-85	0.8015	0.78	0.8234	<.0001
		Age 86-90	0.7243	0.7047	0.7445	<.0001
		Age 91-95	0.6827	0.6599	0.7063	<.0001
		Age > 95	0.6032	0.574	0.6339	<.0001
		Women (REF men)	0.9715	0.9572	0.9861	0.0001
		Black (REF white race)	0.9858	0.9503	1.0226	0.444
		Hispanic	0.9634	0.8905	1.0423	0.3535
		Asian	1.0069	0.9136	1.1097	0.8899
		Other race	0.979	0.9052	1.0588	0.5958
		Medicaid dual-eligible	1.0489	1.021	1.0775	0.0005
		disabled	1.0024	0.9695	1.0363	0.8898
	Admission characteristics	Admitted on weekend	1.0446	1.0188	1.0711	0.0006
		Medium DRG severity (REF low DRG severity)	1.1748	1.1424	1.2082	<.0001
		Highest DRG severity	1.3208	1.2815	1.3613	<.0001
		Cardiac comorbidities	1.4786	1.4184	1.5414	<.0001
		Renal comorbidities	1.6364	1.5922	1.6819	<.0001
		Heme-onc comorbidities	1.119	1.0906	1.1481	<.0001

© 2020 Stevens JP et al. *JAMA Network Open.*

		Neurologic comorbidities	1.1944	1.1628	1.2268	<.0001
		Endocrine comorbidities	1.0088	0.9605	1.0594	0.727
		Rheumatologic comorbidities	1.0483	1.0194	1.078	0.0009
		Pulmonary comorbidities	1.1762	1.148	1.2052	<.0001
Hospital	Case mix of hospital	Q2 hospital mean HCC score (REF Q1, lowest, mean HCC score)	1.0053	0.9559	1.0573	0.8371
		Q3 hospital mean HCC score	1.0549	1.0037	1.1087	0.0353
		Q4 hospital mean HCC score	1.1128	1.0548	1.1739	<.0001
		Q2 hospital mean ICU admissions per year (REF Q1, lowest, mean ICU admissions per year)	1.0431	0.9796	1.1108	0.1877
		Q3 hospital mean ICU admissions per year	1.0355	0.9712	1.1041	0.2858
		Q4 hospital mean ICU admissions per year	1.0502	0.9815	1.1237	0.1559
	Ownership	Government, non-federal (REF not-for-profit)	1.0446	0.9865	1.1062	0.135
		For-profit	1.1106	1.049	1.1759	0.0003
	Teaching status	Major teaching hospital (REF nonteaching hospital)	1.0529	1.0157	1.0915	0.005
		Minor teaching hospital	0.967	0.9312	1.0041	0.081

© 2020 Stevens JP et al. *JAMA Network Open.*

	Region	Rural (Ref urban)	1.5886	1.5208	1.6594	<.0001
		Northeast (REF West)	1.079	0.9709	1.0672	0.461
		Midwest	1.0441	0.9995	1.0905	0.0524
		South	1.025	0.9851	1.0664	0.2226

© 2020 Stevens JP et al. *JAMA Network Open.*

eTable 7. Complete multivariable model for high-consulting hospitalist models, top quartile versus all others, with outcome of visit to a specialist at 90 days.

				95% confidence interval		
			High-consulting	Lower bound	Upper bound	p-value for difference
Odds ratio of visit to a specialist at 90 days			1.069	1.0476	1.0908	<.0001
Patient	Demographic characteristics	Age 71-75 (REF Age < 70)	1.0488	1.0252	1.073	<.0001
		Age 76-80	0.9872	0.9634	1.0115	0.2977
		Age 81-85	0.8021	0.7829	0.8217	<.0001
		Age 86-90	0.6205	0.6048	0.6366	<.0001
		Age 91-95	0.4443	0.4304	0.4585	<.0001
		Age > 95	0.2922	0.2787	0.3062	<.0001
		Women (REF men)	0.8798	0.8668	0.893	<.0001
		Black (REF white race)	0.7721	0.7485	0.7965	<.0001
		Hispanic	1.129	1.0514	1.2125	0.0008
		Asian	1.084	1.0066	1.1673	0.0328
		Other race	0.8958	0.8274	0.9699	0.0066
		Medicaid dual-eligible	0.509	0.4971	0.5212	<.0001
		disabled	0.9224	0.9043	0.9409	<.0001
	Admission characteristics	Admitted on weekend	0.9667	0.9542	0.9794	<.0001
		Medium DRG severity (REF low DRG severity)	0.9698	0.9543	0.9855	0.0002
		Highest DRG severity	0.8643	0.848	0.8809	<.0001
		Cardiac comorbidities	1.5962	1.5639	1.629	<.0001
		Renal comorbidities	1.1691	1.1509	1.1875	<.0001
		Heme-onc comorbidities	1.35	1.3291	1.3712	<.0001

© 2020 Stevens JP et al. *JAMA Network Open.*

		Neurologic comorbidities	0.6037	0.5928	0.6149	<.0001
		Endocrine comorbidities	1.5814	1.5374	1.6267	<.0001
		Rheumatologic comorbidities	1.572	1.545	1.5995	<.0001
		Pulmonary comorbidities	1.095	1.0793	1.1109	<.0001
Hospital	Case mix of hospital	Q2 hospital mean HCC score (REF Q1, lowest, mean HCC score)	1.1621	1.0594	1.2748	0.0015
		Q3 hospital mean HCC score	1.2033	1.0985	1.3181	<.0001
		Q4 hospital mean HCC score	1.2783	1.1637	1.4041	<.0001
		Q2 hospital mean ICU admissions per year (REF Q1, lowest, mean ICU admissions per year)	1.0186	0.9189	1.1291	0.7258
		Q3 hospital mean ICU admissions per year	1.2033	1.0985	1.3181	<.0001
		Q4 hospital mean ICU admissions per year	1.2783	1.1637	1.4041	<.0001
	Ownership	Government, non-federal (REF not-for-profit)	0.9843	0.9083	1.0665	0.6986
		For-profit	0.9419	0.8792	1.0091	0.0885
	Teaching status	Major teaching hospital (REF nonteaching hospital)	1.0402	0.982	1.1019	0.1799
		Minor teaching hospital	0.9154	0.8688	0.9646	0.0009

© 2020 Stevens JP et al. *JAMA Network Open.*

	Region	Rural (Ref urban)	0.4787	0.4495	0.5098	<.0001
		Northeast (REF West)	1.0242	0.9476	1.1071	0.5467
		Midwest	0.8805	0.8191	0.9465	0.0006
		South	0.9522	0.8865	1.0227	0.1791

© 2020 Stevens JP et al. *JAMA Network Open.*

eTable 8. Complete multivariable model for high-consulting hospitalist models, top quartile versus all others, with outcome of mortality at 30 days.

				95% confidence interval		
			High-consulting	Lower bound	Upper bound	p-value for difference
Odds ratio of mortality at 30 days			1.0072	0.9832	1.0319	0.5581
Patient	Demographic characteristics	Age 71-75 (REF Age < 70)	1.1754	1.1337	1.2187	<.0001
		Age 76-80	1.4006	1.3511	1.4519	<.0001
		Age 81-85	1.7583	1.695	1.824	<.0001
		Age 86-90	2.216	2.137	2.2979	<.0001
		Age 91-95	2.8109	2.6998	2.9266	<.0001
		Age > 95	3.6601	3.4801	3.8494	<.0001
		Women (REF men)	0.8546	0.8383	0.8712	<.0001
		Black (REF white race)	0.7828	0.7546	0.8121	<.0001
		Hispanic	0.859	0.7936	0.9297	0.0002
		Asian	0.9417	0.8682	1.0214	0.1473
		Other race	0.8587	0.7974	0.9247	<.0001
		Medicaid dual-eligible	1.0941	1.0685	1.1202	<.0001
		disabled	0.978	0.9491	1.0077	0.1449
	Admission characteristics	Admitted on weekend	1.019	0.9994	1.0389	0.0573
		Medium DRG severity (REF low DRG severity)	3.6664	3.5496	3.787	<.0001
		Highest DRG severity	5.4558	5.2739	5.6439	<.0001
		Cardiac comorbidities	0.9783	0.9481	1.0094	0.17
		Renal comorbidities	1.3844	1.3541	1.4154	<.0001
		Heme-onc comorbidities	1.2571	1.2308	1.2839	<.0001

© 2020 Stevens JP et al. *JAMA Network Open.*

		Neurologic comorbidities	1.4743	1.4427	1.5065	<.0001
		Endocrine comorbidities	0.6715	0.6482	0.6956	<.0001
		Rheumatologic comorbidities	0.7805	0.765	0.7964	<.0001
		Pulmonary comorbidities	0.8904	0.8731	0.9081	<.0001
Hospital	Case mix of hospital	Q2 hospital mean HCC score (REF Q1, lowest, mean HCC score)	0.9841	0.9218	1.0506	0.6313
		Q3 hospital mean HCC score	0.9552	0.8955	1.0188	0.1633
		Q4 hospital mean HCC score	0.962	0.9005	1.0276	0.2499
		Q2 hospital mean ICU admissions per year (REF Q1, lowest, mean ICU admissions per year)	0.8928	0.8172	0.9754	0.012
		Q3 hospital mean ICU admissions per year	0.8885	0.8134	0.9704	0.0086
		Q4 hospital mean ICU admissions per year	0.9369	0.8512	1.0312	0.183
	Ownership	Government, non-federal (REF not-for-profit)	1.0712	1.0073	1.1391	0.0284
		For-profit	1.1115	1.0479	1.179	0.0004
	Teaching status	Major teaching hospital (REF nonteaching hospital)	0.913	0.8678	0.9618	0.0006
		Minor teaching hospital	1.0064	0.9582	1.057	0.7995

© 2020 Stevens JP et al. *JAMA Network Open.*

	Region	Rural (Ref urban)	0.8146	0.7719	0.8596	<.0001
		Northeast (REF West)	0.9242	0.8677	0.9843	0.0143
		Midwest	0.9365	0.8842	0.9919	0.0253
		South	1.0346	0.9805	1.0917	0.2147

© 2020 Stevens JP et al. *JAMA Network Open.*
